# Unlocking the secrets to human NTCP structure

**DOI:** 10.1016/j.xinn.2022.100294

**Published:** 2022-08-01

**Authors:** Xiangbing Qi, Wenhui Li

**Affiliations:** 1 National Institute of Biological Sciences, Beijing 102206, China; 2 Tsinghua Institute of Multidisciplinary Biomedical Research, Tsinghua University, Beijing 100084, China

Sodium taurocholate co-transporting polypeptide (NTCP) is encoded by the solute carrier family 10 member 1 (SLC10A1) gene and is predominantly expressed on the sinusoidal membrane of hepatocytes with the function of hepatic uptake of bile salt, steroid hormones, thyroid hormones, and various bile-acid-conjugated drugs. Almost 10 years ago, NTCP was identified as a functional cellular entry receptor of hepatitis B and hepatitis D viruses (HBV/HDV, respectively),^1^ which are among the major etiological factors leading to cirrhosis, liver failure, and hepatocellular carcinoma and affect around 250 million people worldwide. Previous studies have demonstrated that the binding site of HBV with NTCP is within the first 48 amino acid residues of the N-myristoylated preS1 (myr-preS1) domain of the large envelope glycoprotein. Genetic variations, such as amino acids 84–87 and 157–165 in the NTCP sequences, are responsible for susceptibility to HBV in different species. Individuals who carry the NTCP p.S267F polymorphism on both alleles are associated with increased resistance to chronic HBV infection. Biochemical and virology studies have shown common molecular determinants as well as function separation on the transporting of bile acids and mediating HBV/HDV infection. However, important questions remain unanswered: for example, the binding mode of bile acid or the preS1 lipopeptide to NTCP on the hepatocyte surface, how to differentiate the pathway of substrate uptake and virus infection, and the dynamics of substrate uptake and the molecular mechanisms of virus infection are all unknown.

**Figure 1:**
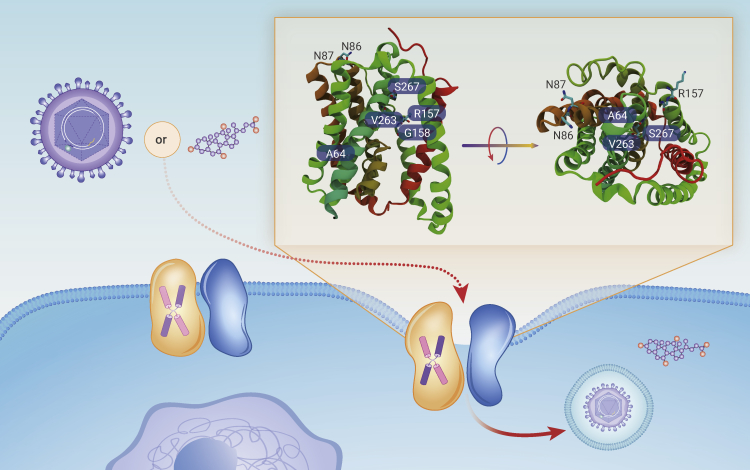
The cryoelectron microscopy structures of NTCP (PDB: 7PQG and 7PQQ). Note: NTCP residue 86 lysine was mutated to asparagine in the structural study.

Remarkably, four landmark publications, based on different approaches, have recently revealed the cryoelectron microscopy (cryo-EM) structure of the human NTCP, providing detailed views of NTCP under different states and conditions (Figure 1). Goutam et al.^2^ reported two cryo-EM maps of NTCP-Nb87 (3.7 Å) and NTCP-Mb91(3.3 Å), respectively, with the aid of the minimum amino acids exchanged, nanobodies (Nb87, Mb91), and the megabody bound to stabilize the conformation. Park et al.^3^ solved the structure of the antibody Fab-bound NTCP complex with a resolution of 3.3 Å and built an atomic model of almost the full length of human NTCP (residues 14–310). Asami et al.^4^ successfully reconstructed cryo-EM maps of human, bovine, and rat NTCPs (Q261A) in a complex with a Fab antibody at resolutions of 3.4, 3.6, and 3.1 Å, respectively. Liu et al.^5^ solved the 2.9 Å resolution cryo-EM structure of human wild-type NTCP bound to NTCP_Fab12 nanobody and disclosed the structural basis of bile salt recognition and transportation.

These published structures clearly show that human NTCP contains nine well-ordered transmembrane α-helices (TM1–9) with the N terminus at the extracellular surface instead of 10 TMs of the bacterial SLC10 homolog (SLC10A2) apical sodium-dependent bile acid transporter (ASBT; from *Neisseria meningitidis* and *Yersinia frederiksenii*). These loop-linked TMs are parallel to the phospholipid bilayer of the membrane and form two kinds of domains: panel (TM1, TM5, and TM6) and core (TM2–4 and TM7–9) domains. These include a highly conserved unwound X-shaped motif, which is constructed by TM3 and TM8 in the middle of the membrane. In complex with different antibodies, NTCP was captured or modeled to two different conformational transitions, and the inward-facing state with a large amphiphilic cavity (molecular surface volume >1500 Å^3^) or the open-pore, outward-facing state with the conformation of the core and panel, which shift approximately 5 Å toward the opposite direction.

According to previous structural studies of SLC10 homologs, the conserved binding sites of Na^+^-1 (S105, N106, S119, T123, and E257) and Na^+^-2 (Q68, E257, T258, C260, and Q261) were predicted by the structural studies and supported by the NTCP-inactivating mutation S267F and A64T, which are close to the sodium-binding sites in the NTCP core domain. Based on the proposed Na^+^ binding mode, the two sodium ions are associated with transporting one bile-acid molecule, and the open-pore state with Na^+^ binding is critical for HBV infection. However, a detailed illustration of the function of these two ions is still limited by the current static state structure. NTCP binding to HBV pre-S1, at least in cell culture conditions, is largely independent of the normal sodium gradient across the cell membrane. This highlights the dynamic and mechanical differences between substrate uptake and HBV translocation.

Based on the reported taurocholate substrate-binding site of the prokaryotic SLC10 homolog, Goutam et al. proposed a substantial structural rearrangement to generate an amphiphilic open pore for bulky molecule integration and translocation. Consistent with the outward-open pore model, Park et al. provided additional dynamic detail of the substrate uptake and speculated that the relative changes in orientation of the core and panel domains created the bile-acid-binding pocket, allowing the gradient of sodium ions to drive substrate uptake. Asami et al. also observed two lipid-like densities inside the hydrophobic tunnel, and one of them occupied a similar pocket of the taurocholate in ASBT *Neisseria meningitidis* (N295, N265, and H294 are in the substrate binding site). However, they are unable to determine whether this site is involved in the substrate translocation pathway due to the interfering co-purified lipids during sample preparation. Liu et al. clearly observed two strong EM density features that were consistent with the polycyclic scaffold of bile salt substrate. Remarkably, based on the high-resolution structure of these two bile salt substrates, they proposed a mechanism of NTCP-mediated bile salt transportation where one bile salt is released to the cytoplasm along with two Na^+^ ions, and another one remains bound to the transporter to prohibit ion leakage.

Accumulated biochemical and mutagenesis studies have established that the HBV entry process is highly sensitive to even a single amino acid variation of NTCP (e.g., A64, K86, N87, K157, G158, V263, or S267) and partially overlaps with the substrate uptake pathway. For instance, the extracellular Lys86 or Asn87 residue of human NTCP is responsible for efficient HBV infection, and the gene editing of three mutants, H84R, T86K, and S87N, with mouse NTCP and human counterparts renders HDV infection in these animals that are otherwise not susceptible to the virus. However, the molecular mechanisms of the underlying HBV interaction with NTCP and virus transposition details are poorly understood.

Goutam et al. analyzed the difference between HBV myr-preS1-GFP binding with the inward-facing state of NTCP-Nb87 and the outward-facing, open-pore state of NTCP-Nb91. They concluded that myr-preS1 preferentially binds to the open-pore state and interacts with exposed interface residues (N262, S267, and L294) between the core and panel domains. Additionally, they mapped the critical NTCP residues (K157-L165) for HBV infections to the extracellular site of TM5 in the panel domain. Besides the direct competitive binding model, Goutam et al. proposed that myr-preS1 could stabilize the outward-facing state of NTCP and suppress conformational changes to the open-to-intracellular side for substrate translocation. This also provided the structural basis for possible HBV blockage via allosteric regulation to the closed, inward-facing conformation of the NTCP.

Park et al. tentatively mapped the possible interaction of residues N9-Q18 of HBV-preS1 with the extracellular loop residues R84-N87 of human (hNTCP) and predicted direct binding between N9 of preS1 with K86 and N87 of hNTCP. These extracellular loop residues are roughly 30 Å from the substrate-binding site of Lys157/Gly158 and were demonstrated to be less relevant to the bile-acid uptake. This indicates a regulatory opportunity for inhibiting viral entry without affecting the innate physiological function of NTCP.

Previous studies have demonstrated that HBV infections are highly myristoylation dependent, and unmodified preS1 has almost 1000-fold lower inhibitory activities than myr-preS1 (half maximal inhibitory concentration of 35 μM versus 39 nM). Additional analysis of the cryo-EM structure of the hNTCP(Q261A), Fab, and myristoyl-preS1 complex by Asami et al. indicated that the myristoyl-preS1 binding site was close to the extracellular surface of the tunnel and adjacent to G158 and S267 of hNTCP. However, the precise position of the myristoyl moiety still cannot be unambiguously identified due to limited resolution.

In combination with the reported biochemical and mutagenesis data, these new studies have uncovered key structural secrets of hNTCP and elucidated the fundamentals of how NTCP controls the bile-acid binding, transporting, and HBV infection. However, the exact dynamics and mechanisms of these steps remain unclear. A large amphiphilic cavity between TM6 and TM9 was observed in the hydrophobic center of the membrane by Goutam et al. In a similar hydrophobic region, Asami et al. proposed that this cavity is lipid filled in a membrane. Alternatively, these structural features could indicate a possible oligomerization site, allowing multiple NTCPs to bind together to form an additional quaternary oligomeric NTCP complex. Final proofs from the future high-resolution structures of oligomeric NTCP or NTCP with bile acids, the myr-preS1, or an optimized domain of L protein containing the preS1 region are required to clarify the binding mode and residue information responsible for the binding and translocation of these distinguished molecules.

Despite the many milestone studies since the discovery of HBV by Baruch Blumberg, an HBV cure is not achievable in most patients with current therapies. Revealing NTCP structure is a great leap toward unlocking the mysteries of NTCP and opening a new era of manipulating NTCP to encourage structure-based anti-HBV drug development or to stem its function in liver diseases. Accumulating evidence has shown that *de novo* infection/reinfection is crucial for sustaining HBV covalently closed circular DNA activity and hence persistent infections in the liver. These advances in our collective understanding of the molecular mechanisms underlying chronic HBV infection mean that efforts to integrate the mechanism of HBV entry blockage into existing therapies could eventually lead to a functional cure for HBV.
